# HOW FRONTLINE STAFF PERCEIVE AND REDUCE GAPS IN KNOWLEDGE AND PRACTICE IN LOCALLY INITIATED QUALITY IMPROVEMENT

**DOI:** 10.1093/geroni/igae098.3512

**Published:** 2024-12-31

**Authors:** B Josea Kramer, Joseph Douglas

**Affiliations:** VA Greater Los Angeles Healthcare System, Los Angeles, California, United States; VA Greater Los Angeles Healthcare System, Los Angeles, California, United States

## Abstract

We evaluated local quality improvement projects that were conducted as part of continuing professional development (CPD) for disciplines that support primary care in Veterans Health Affairs (VA) clinics and medical centers with the goal of understanding how the workforce perceives areas for improvement. The VA Geriatric Scholars Program (GSP) provides core training in its longitudinal CPD through intensive didactic courses in geriatrics, interactive workshop in quality improvement (QI) and coaching to implement a local QI project. Unlike most CPD that includes a QI component in focused training on a specific new clinical intervention, participants in GSP had wide latitude to determine the change to be implemented to enhance healthcare for older Veterans. Using a qualitative approach and applying thematic analysis, we analyzed 740 QI projects at 468 VA facilities undertaken by 809 GSP participants. Clinician-initiated projects indicated the workforce perception of gaps in primary care knowledge and prior training of its interdisciplinary workforce to provide care for older adults in the choice of clinically oriented projects, such as reducing potentially inappropriate medications or instituting fall prevention activities. Other projects focused on enhancing processes within the healthcare system, such as timely lab reports or referral protocols. Our results indicate that educational needs assessments can be conducted at the interprofessional team level, identifying the needs for CPD that are perceived as relevant by the workforce.

